# Tackling the 3 Big Challenges Confronting Health Technology Assessment Development in Asia: A Commentary

**DOI:** 10.1016/j.vhri.2019.07.001

**Published:** 2020-05

**Authors:** Yot Teerawattananon, Yik Ying Teo, Saudamini Dabak, Waranya Rattanavipapong, Wanrudee Isaranuwatchai, Hwee-Lin Wee, Nan Luo, Alec Morton

**Affiliations:** 1 Health Intervention and Technology Assessment Program, Ministry of Public Health, Nonthaburi, Thailand; 2 Saw Swee Hock School of Public Health, National University of Singapore, Singapore; 3 Institute of Health Policy, Management and Evaluation, University of Toronto, Toronto, Canada; 4 Department of Management Science, University of Strathclyde, Glasgow, England, UK

**Keywords:** health technology assessment, HTA, HTA in Asia, priority setting

## Abstract

There has been continuous development in the field of health technology assessment (HTA) owing to the added value of HTA in supporting healthcare reimbursement decisions. Collaboration and engagement among countries in Asia has been carried out to share experiences and learning on the barriers and factors facilitating the implementation and use of HTA in policy making. A symposium on the topic of *Health Technology Assessment (HTA): Selecting the Highest Value Care* was held on January 10, 2019 at the National University of Singapore, during which 3 major challenges confronting HTA development in Asia were identified. The symposium also offered possible ways to overcome the challenges.

## Highlights

• Countries in Asia increasingly recognize and adopt health technology assessment (HTA) in setting priorities for healthcare. Nevertheless, 2 common challenges can be identified, namely, lack of infrastructure and technical capacity to cope with the increasing demand for HTA, and the inadequate involvement of stakeholders in the HTA process.
• Solutions identified at the HTA symposium in Singapore to overcome these challenges were that countries should implement a tailored and transparent mechanism for using HTA, promote existing HTA capacity, and strengthen the understanding of HTA among the stakeholders.
• Health technology assessment symposia such as the one organized in Singapore provide a platform for countries to share knowledge and challenges, and bring different views to build into something greater and overcome the challenges. Such symposia should be organized regularly.

## Introduction

On January 10, 2019, a symposium on the topic of *Health Technology Assessment (HTA): Selecting the Highest Value Care* was held at the National University of Singapore (NUS).^1^ There were approximately 300 participants from more than 10 countries, including Ghana, India, Indonesia, Malaysia, the Philippines, Singapore, and Thailand, in addition to the Hong Kong Special Administrative Region of the People’s Republic of China. Speakers included the chair of the Subcommittee of the National List of Essential Medicines in Thailand and the HTA Committee in Indonesia in addition to academics and representatives from global partners such as the International Decision Initiative (iDSI) and the World Health Organization.

Asia is viewed as a region where interest and capacity in HTA has significantly improved over a short period. Nevertheless, the countries that have implemented HTA are at various levels of economic development and stages of implementing public health insurance schemes. During the symposium, important issues on the role of HTA in healthcare were discussed and common characteristics and different points of view across countries were observed. In this article, the authors would like to highlight 3 challenges that emerged during the symposium: (1) the increasing need and demand for health priority setting in this region, (2) the lack of infrastructure and technical capacity to cope with the increasing demand, and (3) the inadequate involvement of stakeholders in the HTA process. We then propose the way forward for HTA in the region.

## Need and Demand for HTA

Research suggests that the increase in healthcare spending is due to economic development, adoption of high-cost technologies, an aging population, and rapid uptake of universal health coverage (UHC).^2^, ^3^, ^4^ This movement, in turn, has led to an increase in the demand for priority setting using HTA. Although these trends are sometimes presented as problems, this symposium takes a different view and highlights that these are, in fact, the byproducts of our success in the spheres of social and economic development in recent decades. These complex problems exist, and HTA represents an approach to support complex decision making, for example, to better inform decision makers to make tradeoffs in resource allocation.

The advancement in science and technology has made more health technologies available in the market. This trend poses a challenge not only to the government or payer who needs to decide whether a new technology is to be added in the UHC benefits package, but also to health professionals who want to adopt the best clinical practice and to the patients who may recognize that better treatment is available but not accessible. Second, people are now living healthier and longer lives, which results in a greater demand for health services. Lastly, good governance has become more established in health systems, and that has promoted the use of evidence-informed policy decisions.

To respond to the aforementioned challenges, many countries in the Asian region use HTA for priority setting, although the mode of implementation depends on the health system design and local social values. For example, in India, state governments play a major role in implementing healthcare policies, and the private sector is a significant provider of services and households bear a high proportion of healthcare costs. Meanwhile, Thailand has a centralized system with a high proportion of government spending on healthcare. Indonesia presents a different case where there is a focus on the devolution of decision making to local governments. In Singapore, there is an emphasis on personal responsibility, where individuals are expected to contribute to the cost of healthcare. These examples reflect the heterogeneity in the healthcare systems in the region and the challenge of tailoring HTA in a way that is responsive to local circumstances. The difference calls for governments or payers to design a mechanism for using HTA for healthcare decision making that is relevant to the contextual factors, and for HTA agencies to identify the role and opportunities to provide the highest value for the system.

## HTA Infrastructure

Representatives from most countries at the symposium expressed concern about the lack of infrastructure for HTA, such as reliable cost databases and local health outcomes data to estimate quality-adjusted life years. Further, there is a lack of technical capacity, especially in the field of health economics and disease modelling, and the increased need and demand for HTA exacerbates the gap between demand and supply for HTA technical capacity. The Health Intervention and Technology Assessment Program (HITAP) has conducted annual training for local scholars for the past 15 years, but that alone is not enough to meet demand in the region. The NUS is now trying to bridge this gap in the region by developing an annual course on HTA to supplement local training in countries through the newly established Center for Health Intervention and Policy Evaluation Research. Nevertheless, the question remains of how such initiatives can be scaled up. Suggestions from the symposium called for making greater efforts to strengthen HTA capacity through regional and global collaborations such as HTAsiaLink,^5^ and building on the existing work under iDSI, such as the iDSI Reference Case^6^ and the Guide to Economic Analysis and Research. The Guide to Economic Analysis and Research, an online resource funded by iDSI, shows that there are 43 HTA guidelines available worldwide, yet only 3 are in low- and middle-income countries.^7^ If all countries were to have their own guidelines, this would mean developing more than 100 new guidelines. The authors believe that this need not be the way forward and the investment could be more efficiently applied, if countries learn from one another on these infrastructural and technical aspects without having to reinvent the wheel.

During the last decade, academics and HTA practitioners have paid more attention to the generalizability and transferability of methodological aspects of HTA and its results.^8^, ^9^, ^10^, ^11^ In this decade, the HTA community ought to pay at least equal attention to the generalizability and transferability of HTA applications. This would allow meaningful conclusions on why and how HTA is being used in different countries. For example, in Thailand, HTA is used for assessing new technologies that are to be introduced to the benefits package, whereas in Singapore, HTA is being used to make subsidy decisions for existing technologies. In India, HTA is being used to develop a benefits package for its UHC program, whereas Indonesia is using HTA to delist less cost-effective medicines and interventions from its benefits package. Better understanding of how HTA is used in practice will provide a stronger foundation for performance comparison and benchmarking of HTA by tracking the progress of HTA systems in each country, as well as systematic information sharing and collaboration on technical studies through, for example, joint HTAs for interventions and maintaining repositories of existing relevant models that are of common interest.

## Stakeholders

There was consensus at the symposium that not only technical capacity but also the institutional arrangements are critical to ensuring a credible process for incorporating evidence into policy with transparency and participation as core principles. This is where the role of stakeholders becomes significant. There was wide-ranging discussion of the question of whether the current practice is sufficient for identifying impactful topics for HTA through stakeholder participation and how to ensure that assessments are not driven by supply but rather by need and demand. In particular, the role of different stakeholders, for example, private sector and civil society, may differ at different stages of HTA development.

The authors propose the following way to take HTA forward in Asia. It was agreed that enhancing the understanding of HTA among various groups of stakeholders and augmenting their involvement will be crucial in the coming years. The creation of the National Institute for Health and Care Excellence and HITAP brought these issues in the public realm and allowed stakeholders to debate on health policies.^12^, ^13^ For example, when the National Institute for Health and Care Excellence says no to including medicines, or when HITAP advises against inclusion of new technologies, it provokes debate about these issues among health professionals, decision makers, and media and sensitizes the public about the rationale for rationing by decision makers. Furthermore, there are several communication methods HTA agencies can implement, such as inviting relevant stakeholders to participate in consultation meetings in the HTA process to facilitate direct interaction, or setting up webpages dedicated to disseminating HTA results.

Based on the discussion during the symposium, the authors believe that it is the responsibility of HTA agencies to better communicate the use of HTA and its societal benefits.

## Conclusion

Table 1 summarizes the recommendations that address the main challenges. The HTA symposium in Singapore has ended, but the earlier discussion forebodes the beginning of a new chapter of HTA for Asia. The authors expect that successfully addressing the 3 challenges will inform the future directions of HTA in the region and beyond.

**Table 1:** Recommendations for successful HTA to policy making in Asia.

Challenges	Recommendation
Increasing need and demand for HTA	To design a tailored and transparent mechanism for using HTA
Lack of infrastructure and technical capacity to cope with the increasing demand	To focus on building on existing capacity, that is, HTA public goods and network, and creating a better wheel (not to reinvent one)
Inadequate involvement of a broad range of stakeholders, including public authorities, healthcare providers, payers, academic, industry, citizens and patients, media	To enhance the understanding of HTA among the public and stakeholder involvement in HTA process

HTA indicates health technology assessment.
